# Mapping digital health maturity models and accreditation-linked standards: a scoping review to position the National Accreditation Board for Hospitals & Healthcare Providers (NABH) digital health standards of India

**DOI:** 10.1186/s12913-026-14301-y

**Published:** 2026-03-11

**Authors:** Margeyi Mehta, Jigish Shah, Urvish Joshi, Sharon Baisil, Sanjay Kini B.

**Affiliations:** 1 Department of Clinical Biochemistry, Medical College Baroda, The Maharaja Sayajirao University of Baroda, Vadodara, Gujarat India; 2 Department of Microbiology, Parul Institute of Medical Sciences & Research, Parul University, Vadodara, Gujarat India; 3 Department of Community Medicine, Narendra Modi Medical College, Gujarat University, Ahmedabad, Gujarat India; 4 Department of Community Medicine, Malankara Orthodox Syrian Church Medical College, Kerala University of Health Sciences, Kolenchery, Kerala India; 5 Department of Community Medicine, Kasturba Medical College, Manipal Academy of Higher Education, Manipal, India

**Keywords:** NABH Digital Health Standards, Digital health maturity model, Healthcare accreditation, Scoping review, Digital health governance, Health Information Systems (HIS), Interoperability, AI governance, Cybersecurity, Health equity

## Abstract

**Background:**

Digital transformation in healthcare is guided globally by digital health maturity models and accreditation-linked standards. In India, the National Accreditation Board for Hospitals & Healthcare Providers (NABH) introduced landmark Digital Health Standards (DHS) for hospitals (2025 draft). This provides a national framework, but its positioning within the complex global landscape is unclear, making it challenging for stakeholders to benchmark progress and plan strategically.

**Objectives:**

The primary objective was to map the features, domains, and assessment approaches of prominent international digital health maturity models and accreditation-linked standards. The secondary objective was to conduct a comparative analysis of these frameworks against the NABH-DHS to identify areas of convergence, divergence, and critical gaps.

**Methods:**

A scoping review adhering to PRISMA-ScR guidelines was conducted. Peer-reviewed databases (PubMed, Embase, Scopus) and grey literature sources were searched from inception to 26 July 2025. We included sources describing national or international maturity models or accreditation standards for healthcare provider organizations. Data were charted using a customized form, synthesized narratively (SWiM), and comparatively analysed using a conceptual crosswalk matrix and thematic gap map.

**Results:**

38 sources were included, comprising systematic/scoping reviews (*n* = 8), official reports/standards (*n* = 17), and primary studies (*n* = 13). Key international frameworks mapped include HIMSS EMRAM, NHS England’s WGLL, WHO-PAHO IS4H, JCI, and Australian models. The NABH-DHS (structured across 8 chapters) shows strong alignment with these frameworks in core domains: Leadership, Governance, Clinical & Patient Safety, and Information & Data Management. However, the comparative analysis identified emerging gaps vis-à-vis global best practices. These include absent or minimal coverage for explicit AI governance, advanced cybersecurity maturity (e.g., alignment with NIST CSF 2.0), granular interoperability maturity assessment, a dedicated health-equity lens, and the systematic integration of Patient-Generated Health Data (PGHD).

**Conclusions:**

The NABH-DHS provide a robust and comprehensive foundation for core digital assurance in India, converging well with international best practices on foundational elements. Our mapped findings are presented as external policy guidance. We recommend that future NABH revisions incorporate pragmatic, light-weight requirements to address the identified gaps (AI governance, advanced cybersecurity, interoperability metrics, and equity). This can be achieved through annexes or tiered ‘Digital Plus’ badges that reference mature external frameworks (e.g., ISO/IEC 42001, NIST CSF 2.0), ensuring a future-proof, phased implementation that safeguards patient trust as the Indian digital health ecosystem matures.

**Supplementary Information:**

The online version contains supplementary material available at 10.1186/s12913-026-14301-y.

## Introduction

Digital transformation in healthcare extends beyond digitizing paperwork; it entails rethinking and redesigning care pathways, workflows, and organizational models enabled by data and technology to improve outcomes, safety, and efficiency. This transition promises to enhance clinical outcomes, improve patient safety, and optimize operational efficiency [1]. To guide and measure this complex transformation, two key instruments have emerged globally: digital health maturity models and accreditation-linked digital standards. Maturity models, such as the widely adopted Healthcare Information and Management Systems Society (HIMSS) Electronic Medical Record Adoption Model (EMRAM), provide a staged roadmap for healthcare organizations to advance their digital capabilities [2]. Accreditation standards, enforced by bodies like The Joint Commission International (JCI), integrate digital health requirements into broader quality assurance and patient safety frameworks, making digital proficiency a mandatory component of quality care [3]. 

In India, the National Accreditation Board for Hospitals & Healthcare Providers (NABH), a constituent board of the Quality Council of India (QCI), has taken a landmark step to formalize digital health within its quality paradigm. NABH has released draft Digital Health Standards (DHS) for Hospitals and complementary Standards for Hospital Information System (HIS) and Electronic Medical Record (EMR) Systems that were first circulated in 2023–2024 and are currently available online as 2025 draft documents [4]. We refer to these collectively as the NABH-DHS and focus here is on the hospital-facing requirements [5]. The hospital standards are structured across eight chapters-Leadership; Governance; Clinical & Patient Safety; Patient Engagement; Information & Data Management; Technology & Infrastructure; Risk Management; and Human Resource Management-encompassing 38 standards and 181 objective elements. These are tiered into three maturity levels (Silver, Gold, Platinum) to provide a phased implementation pathway [1]. The draft HIS/EMR standards complement this by defining technical and functional requirements for software systems themselves [4]. We note that NABH public materials describe the Digital Health Standards by chapter in more than one way across releases. For example, the current Frequently Asked Questions (FAQ) groups eight chapters as Access and Continuity of Care, Care of Patients, Medication Management, Digital Infrastructure, Digital Operations Management, Finance and Procurement, Human Resource Management, and Information Management Systems, whereas earlier/draft texts reference chapters aligned to governance, clinical and patient safety, patient engagement, information and data management, technology and infrastructure, risk management, and Human Resource (HR) management [5]. To remain internally consistent, we use the chapter labels reported in the primary NABH standards documents referenced for this review and cross-reference to the FAQ descriptors (in Table 3 footnotes).

Despite the comprehensive nature of these new NABH standards, their introduction raises a critical question for Indian healthcare stakeholders: How do these standards align with established international models and best practices? Hospital administrators, Chief Information Officers (CIOs), and Health-Information Technology (IT) vendors currently lack a synthesized evidence map that positions the NABH framework against global counterparts. This absence makes it challenging to benchmark progress, identify unique Indian requirements, and strategically plan for future-state digital capabilities that are both locally relevant and globally aligned.

This scoping review aims to address this gap. The primary objective is to systematically map the key features, domains, and assessment approaches of prominent international digital health maturity models and accreditation-linked standards. The secondary objective is to conduct a comparative analysis of these frameworks against the NABH-DHS to identify areas of convergence, divergence, and critical gaps [5]. 

The review is guided by the Joanna Briggs Institute (JBI) guidance on Population, Concept, and Context (PCC) framework specification for scoping reviews where Population was healthcare provider organizations and health systems, Concept was digital health maturity models and accreditation standards and Context was global, with a specific comparative focus on the Indian NABH standards [6]. 

## Methods

### Protocol and registration

We followed Arksey & O’Malley as refined by JBI, with protocol registered on Open Science Framework (OSF) with DOI:10.17605/OSF.IO/39J8K [6–8].

### Reporting guideline

Reporting adheres to PRISMA‑ScR, with narrative synthesis informed by Synthesis Without Meta-analysis (SWiM) where applicable [9, 10]. 

### Eligibility criteria

We included sources describing a maturity model, assessment framework, or accreditation standard focused on digital capabilities of healthcare provider organisations; including systematic/scoping reviews, primary studies, and official documents from standards‑setting bodies; Search was limited to English‑language only.

### Information sources

MEDLINE (PubMed), Embase, and Scopus were searched from database inception to 26 July 2025. Structured grey-literature searches happened between 10 and 26 July 2025. In total, these structured grey-literature searches yielded 42 unique records across the NABH/QCI portals (including the NABH-DHS microsite), World Health Organization - Pan American Health Organization- Information Systems for Health (WHO/PAHO IS4H) pages, Healthcare Information and Management Systems Society (HIMSS) maturity-model resources (including Electronic Medical Record Adoption Model -EMRAM), National Health Service (NHS) England’s ‘What Good Looks Like’ (WGLL) guidance pages, Joint Commission International (JCI) materials, Australian Commission on Safety and Quality in Health Care (ACSQHC) and National Safety and Quality Health Service Standards (NSQHS) digital health and safety resources, the Office of the National Coordinator for Health Information Technology (ONC) (from U.S. Department of Health and Human Services) Interoperability Standards Advisory, and Koita Foundation reports [2, 3, 5, 11–14]. Because records were de-duplicated across portals at the screening stage and several items were accessible through more than one site, we report only the total grey-literature yield rather than per-portal counts. We did not retain stable per-portal counts at the pre-deduplication stage; hence only the consolidated grey-literature total (*n* = 42) is reported which may slightly limit transparency on the relative contribution of each source. Final PubMed/Embase/Scopus searches were executed on 24–26 July 2025; grey sources were last checked on 26 July 2025. Reference lists were scanned.

### Search strategy

The operational PubMed query was: (‘digital health’[Title/Abstract] OR ‘digital maturity’[Title/Abstract] OR ‘digital health maturity’[Title/Abstract] OR ‘maturity model’[Title/Abstract] OR ‘maturity assessment’[Title/Abstract]) AND (‘health’[MeSH Terms] OR ‘health information systems’[MeSH Terms] OR ‘medical informatics’[MeSH Terms]). The alternate formulation shown in Supplement (Title/Abstract + MeSH) returned the same candidate set in our pilot and is retained for transparency (Supplement S1). Similarly, Embase and Scopus databases were also searched. Google/Google scholar was used to search grey literature. The reference lists of included articles were also manually scanned for additional relevant sources. (Detailed search strategy as per Supplementary File S1)

### Study selection and de-duplication

**Fig. 1:**
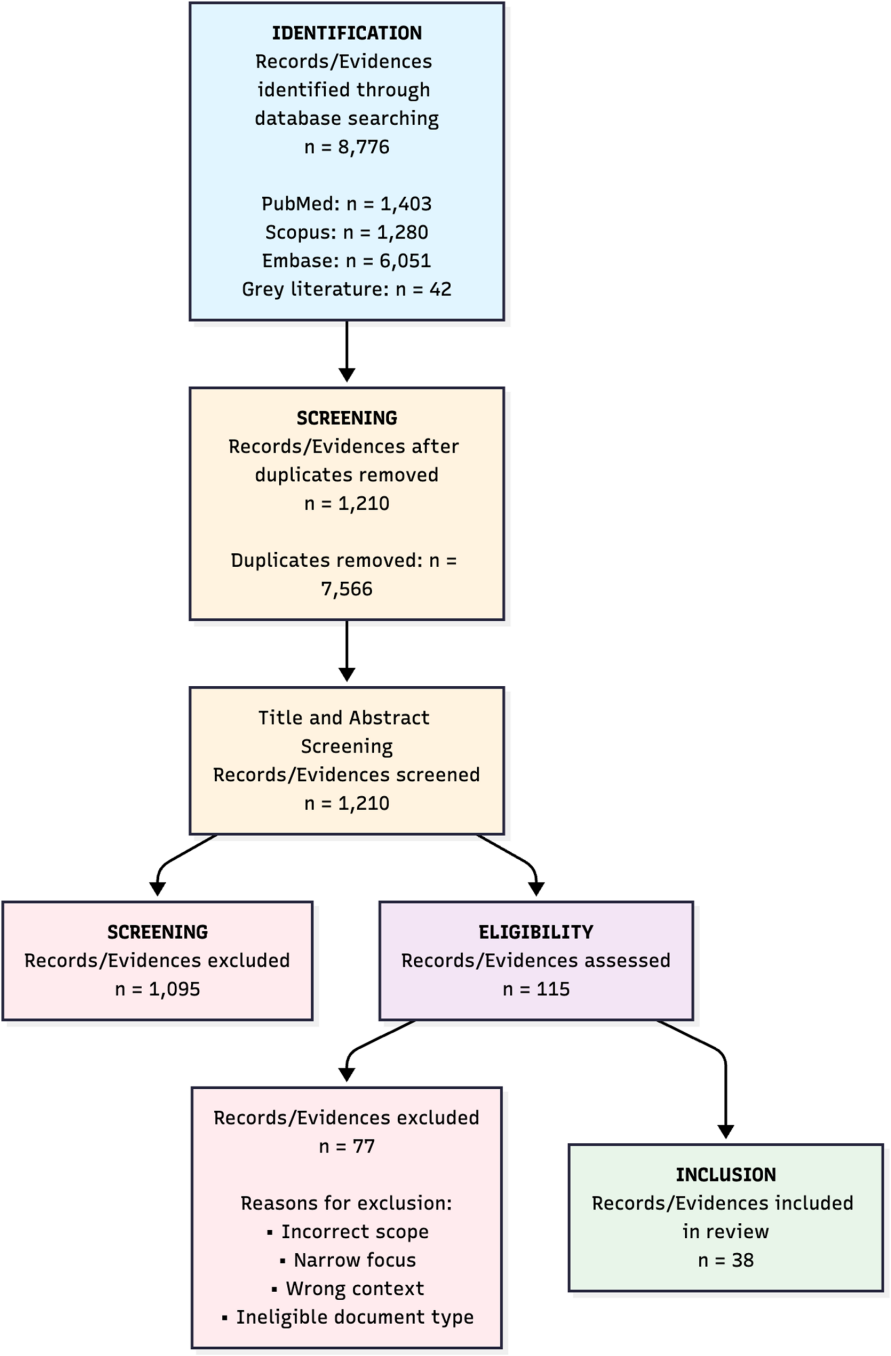
PRISMA-ScR flow diagram

Rayyan was used for screening and de‑duplication [15]. Records were imported into Rayyan; automated and manual duplicate detection were applied, with reviewer verification before title/abstract screening. Conflicts on screening decisions were resolved by discussion between two reviewers (MM, JS) with adjudication by a third reviewer (UJ) where required. Reasons for exclusion at full text screening level are reported in Fig. 1 as PRISMA‑ScR flow diagram.

### Data charting and mapping rules

A piloted form extracted the model/standard name; originating body and country; objectives; structure (domains, stages/levels); assessment method; evidence of validation; and links to accreditation or national policy.

### Crosswalk mapping procedure

Two reviewers independently mapped each NABH chapter to domains of HIMSS EMRAM, NHS WGLL, and PAHO IS4H using pre-specified anchors: (i) explicit domain/criterion overlap; (ii) functional equivalence (e.g., patient engagement ↔ citizen empowerment); and (iii) evidence of regulatory use (e.g., Continuous Quality Control - CQC use of WGLL). Alignment strength was rated ‘strong’ when ≥ 2 anchors were met with direct textual coverage; ‘partial’ when only one anchor was met or coverage was implicit. Disagreements were resolved by consensus with a third (UJ) and fourth (SB) reviewer. External policy frameworks (e.g., National Institute of Standards and Technology Cybersecurity Framework - NIST CSF 2.0; International Organization for Standardization - ISO and the International Electrotechnical Commission - IEC − 42001; Interoperability Maturity Toolkit) were not used to score alignments; they were referenced later to contextualize observed gaps [2, 5, 11, 12, 16–18]. 

### Critical appraisal

As per JBI guidance for scoping reviews, a formal critical appraisal of individual sources for risk of bias was not conducted, as the aim is to map the extent and nature of the evidence rather than synthesize a quantitative effect estimate [8]. 

### Synthesis plan

We produced a descriptive numerical summary, a narrative synthesis, a crosswalk matrix aligning international domains with NABH chapters, and a thematic gap map. Narrative synthesis followed SWiM guidance; descriptive statistics summarize sources by type/origin; crosswalk matrices depict alignment; a gap map visualizes under-represented areas [10]. Priority gap domains were identified by triangulating NABH chapters with weak or absent coverage in the crosswalk and themes that were repeatedly emphasized in at least two ‘frontier’ frameworks or toolkits (e.g., NIST CSF 2.0, the Health Information Systems Interoperability Maturity Toolkit, and ISO/IEC 42001), as well as in recent syntheses on digital equity, Artificial Intelligence (AI) governance and patient-generated health data [16, 17]. 

## Results

### Study selection

We identified 8,776 records (databases = 8,734; grey = 42). After 7,566 duplicates were removed in Rayyan, 1,210 records were screened; 115 full texts were assessed. During the full-text assessment, 77 sources were excluded primarily due to incorrect scope (not presenting a structured maturity model or assessment framework), narrow focus (specific to a single technology like telehealth), wrong context (designed for non-healthcare provider settings), or ineligible document type (e.g., editorial, commentary, or news report lacking detailed methodology). Ultimately, 38 sources met the eligibility criteria and were incorporated into the narrative synthesis. The PRISMA-ScR flow diagram is presented in Fig. 1.

### Characteristics of included sources

Of the 38 included sources/records, 8 were systematic/scoping reviews; 17 were official reports or standards; and 13 were primary studies or descriptions of specific models/tools. Sources originated from North America, Europe, Australia and international bodies, with several tailored to LMIC contexts. Table 1 included all selected sources (*n* = 38) with the framework/standard they inform.

**Table 1:** Included sources with framework/standard mapping (*n* = 38)

ID #	Source (short title)	Type	Originating body & region	Year	What it assesses (unit)	Structure / Levels	Assessment method/tool	Primary domain(s)	Notes / NABH crosswalk cue
1	HIMSS EMRAM [2]	Model	HIMSS - Global	2025	Hospital digital maturity	8 stages (0–7)	On‑site validation (higher stages)	Infrastructure, EHR, decision support, interoperability, patient engagement	Aligns across NABH Ch.3,5,6; Stage‑7 ↔ leadership analytics (Ch.1)
2	NABH Digital Health Standards [5]	Accreditation standard	NABH - India	2025	Hospital digital health capability for accreditation	8 chapters, 38 standards, 181 objective elements; Silver/Gold/Platinum	External accreditation survey	Leadership, governance, safety, data, tech, risk, HR, patient engagement	Core comparison anchor
3	NABH HIS/EMR Standards [4]	Standard (technical/functional)	NABH - India	2025	HIS/EMR system requirements	Chapters with functional/technical criteria	Documentation review & facility assessment	Information management, security, interoperability	Complements NABH digital health standards
4	NHS ‘What Good Looks Like’ (WGLL) [12]	Capability framework	NHS England - UK	2025	Provider‑level digital capability	7 success measures	Regulator‑used rubric (CQC)	Well‑led, smart foundations, safe practice, support people, empower citizens, improve care, healthy populations	Crosswalk driver for Ch.1–6
5	PAHO/WHO IS4H Maturity Model [11]	Model	PAHO/WHO - Americas/Global	2025	National & provider IS maturity	5 maturity levels across domains	Self‑assessment toolkit	Leadership & governance; data mgmt.; technology	Maps to NABH Ch.1,2,5,6
6	NSQHS Standards (2nd ed., 2021 update) [13]	Accreditation standard	ACSQHC - Australia	2025	Hospital quality & safety incl. digital	8 standards, action items	External accreditation survey	Clinical governance, medication safety, communication, information management	Supports patient safety & information management
7	NHS Digital Maturity Assessment (DMA) [20]	Programme/assessment	NHS England - UK	2025	Trust digital maturity	Multi‑domain scoring against WGLL	Self/assessor scoring	Foundations, EPR, interoperability, user experience	Operationalises WGLL
8	Data Security and Protection Toolkit (DSPT) [21]	Compliance framework	NHS England - UK	2025	Org. data security compliance	Assertion‑based standards	Annual self‑assessment + audits	Security, IG, privacy	Aligns with NABH Ch.7 (risk)
9	JCI Accreditation Standards (Hospitals, 7th ed.)	Accreditation standard	JCI - Global	2025	Hospital quality & safety (info mgmt. embedded)	Chapters and measurable elements	External accreditation survey	Management of Information; patient safety; privacy	Crosswalk for Ch.3,5,7
10	JCI Telehealth Certification Standards [3]	Certification standard	JCI - Global	2025	Telehealth service quality & safety	Domains/requirements (selected)	External review/certification	Governance, clinical safety, security, continuity	Extends digital safety to virtual care
11	National Institute for Health and Care Excellence (NICE) Evidence Standards Framework (ESF) [22]	Standards framework	NICE - UK	2025	Evidence & impact standards for Digital Health Technologies (DHTs)	Tiered evidence levels by function/risk	Evidence submission & review	Clinical effectiveness, economic impact, data	Guides appraisal of apps/tools intersecting NABH Ch.3,4,5
12	NICE ‘How to meet the ESF’ [22]	Guidance	NICE - UK	2025	Compliance with ESF	Practical checklists	Self‑assessment	Study design, outcomes, usability	Implementation support
13	ONC Interoperability Standards Advisory (ISA) [14]	Advisory catalogue	ONC - USA	2025	Interoperability standards landscape	Thematic sections	Reference use	Messaging, vocabularies, APIs	Supports NABH Ch.6,5
14	Scotland Digital Maturity Assessment (SDMA)(2023) [23]	Programme/report	Scottish Govt/ Convention of Scottish Local Authorities (COSLA)	2025	National provider maturity results	Multi‑domain	Self‑assessment (national rollout)	Strategy, data, tech, workforce	Comparative national benchmark
15	MEASURE HIS Interoperability Maturity Toolkit [18]	Maturity toolkit	MEASURE Evaluation	2025	Interoperability maturity (health info systems)	Levels per capability	Self‑assessment toolkit	Governance, standards, processes, measurement	Fills NABH gap on interoperability metrics
16	NIST CSF 2.0 [16]	Framework	NIST - USA	2025	Org. cybersecurity maturity	CSF functions & tiers	Self/3rd‑party assessment	Identify‑Protect‑Detect‑Respond‑Recover‑Govern	Deepens NABH Ch.7 (risk)
17	WHO Guideline: Digital Interventions [24]	WHO guideline	WHO - Global	2025	Policy/implementation guidance	Recommendations	Guideline adoption	Governance, equity, data use	Context for governance & equity
18	PAHO IS4H Toolkit - Planning Guide [11]	Toolkit	PAHO - Americas	2025	Plan & execute maturity assessment	Process guidance	Toolkit‑based	Governance, strategy, investment	Complements IS4H model
19	Australian Digital Health Literacy (ADHA) Workforce & Education Roadmap [43]	Roadmap	Australian Digital Health Agency	2025	Workforce capability development	Priority areas	Programme/roadmap	Workforce skills, education	Aligns with NABH Ch.8 (HR)
20	J Med Internet Res - Sylla et al. (2025) [25]	Review	Journal of Medical Internet Research	2025	25 years of digital health in Low-and-middle-income-countries (LMICs)	Rapid systematic review	Literature synthesis	Scale‑up, sustainability	LMIC lens
21	Health Policy & Planning (HPP) - Dharmagunawardene et al. (2025) [26]	Scoping review	HPP	2025	Accreditation programmes in LMICs	Scoping review	Literature synthesis	Standards adoption, readiness	Accreditation context
22	Food and Drug Administration (FDA) Guidance - Real-world data (RWD) from EHR/Claims (2025) [27]	Guidance	U.S. FDA - USA	2025	Use of RWD for drugs	Guidance	Review‑based	Data quality, provenance	Supports Patient-Generated Health Data (PGHD)/RWD integration gap
23	FDA Guidance - Real World Evidence (RWE) for Devices (2025) [27]	Guidance	U.S. FDA - USA	2025	RWE for devices	Guidance	Review‑based	Data sources, validity	As above
24	International Medical Device Regulators Forum (IMDRF) Registry Usability Tools (2025) [28]	Tools	IMDRF - International	2025	Registry usability for regulation	Tooling criteria	Self‑/peer‑ assessment	Data quality, linkage, governance	Registry maturity metrics
25	Front Public Health - UK Disabilities (2025) [29]	Scoping review	Front Public Health	2025	Telehealth & disability (UK)	Scoping review	Synthesis	Accessibility, UX	Accessibility standards cue
26	JCI Telehealth Certification (web) [42]	Programme page	JCI - Global	2025	Programme overview	Web criteria	External audit	Governance, clinical quality, security	Confirms certification scope
27	ISO/IEC 42,001 - AI Management System Requirements [17]	Standard	ISO/IEC - International	2025	Organizational AI management system	Management system (requirements)	Certification/audit against standard	Governance; risk; lifecycle; ethics	Context for AI governance gap; maps to NABH Ch.1 (Leadership), Ch.2 (Governance), Ch.7 (Risk)
28	World Bank - Digital‑in‑Health (2025) [30]	Policy report	World Bank - Global	2025	Prioritise–Connect–Scale approach	Roadmap	Policy uptake	Governance, financing, scale	Implementation context
29	Australian Council on Healthcare Standards (ACHS) - Evaluation and Quality Improvement Programme (EQuIP) Accreditation (overview) [31]	Accreditation programme	ACHS - Australia	2025	Hospital/service accreditation	Standards & criteria (EQuIP; NSQHS-aligned)	External accreditation survey	Clinical governance; quality improvement; information management	Distinct accreditation body; complements NSQHS content; maps to NABH Ch.1–3,5,7
30	Telemedicine & e‑Health - India Disabilities (2024) [32]	Scoping review	Telemed e‑Health	2024	Telehealth equity in India	Scoping review	Synthesis	Equity, access	Equity lens for NABH Ch.4
31	JAMIA Critical Thematic Review - Liaw & Godinho (2023) [33]	Review	JAMIA	2023	Digital health & capability maturity models	Thematic synthesis	Literature synthesis	Capability domains, journeys	Consolidates provider‑focused CMMs
32	JCI Journal - Telehealth Safety Infrastructure (2023) [42]	Primary study/commentary	The Joint Commission Journal on Quality and Patient Safety (JQPS)	2023	Safer, equitable telehealth infra	Conceptual framework	-	Equity, safety, continuity	Virtual‑care safety linkage
33	NPJ Digital Medicine - Richardson et al. (2022) [34]	Framework	NPJ Digit Med	2022	Digital health equity framework	Domains (individual‑system)	-	Equity, governance, measurement	Equity metrics cue
34	Ann Fam Med - Communication Gaps – Timmins et al. (2022) [35]	Primary study	Ann Fam Med	2022	Care transitions communication	Observational	-	Interoperability outcomes	Outcome rationale for maturity targets
35	Health Policy & Planning - Mansour et al. (2020) [36]	Review	HPP	2020	Hospital accreditation development in LMICs	Narrative review	Literature synthesis	Accreditation pathways	Accreditation context
36	NICE ESF context - Veinot et al. (2018) [37]	Perspective	JAMIA	2018	Equity risks of informatics interventions	Conceptual	-	Equity, unintended harms	Equity lens for patient engagement
37	JMI Systems Maturity - Carvalho et al. (2016) [38]	Review	Journal of Medical Systems	2016	HIS/HIT maturity model landscape	Narrative categories	Literature synthesis	IS maturity domains	Background synthesis (included review)
38	HIR Review - Luna et al. (2014) [39]	Review	Healthcare Informatics Research	2014	LMIC informatics implementation challenges	Narrative	Literature synthesis	Sustainability, governance, infrastructure	LMIC context cues for crosswalk

Table 2 summarises the key characteristics of the most prominent frameworks identified. Table 2 also foregrounds the six frameworks most frequently cited and/or with clear national/regulatory uptake (HIMSS EMRAM; NHS WGLL; PAHO/WHO IS4H; NABH DHS; JCI; Australian ADHCF), with the full list and linkages to included sources provided in Table 1.

**Table 2:** Characteristics of included digital health frameworks

Framework / Standard	Originating Body	Country / Region	Core Objective	Structure & Assessment
NABH-DHS [4]	NABH	India	To accredit and guide hospitals on digital health implementation for quality and safety.	8 chapters, 38 standards, 181 objective elements. Assessed at Silver, Gold, Platinum maturity levels.
HIMSS-EMRAM [2]	HIMSS	USA / Global	To guide hospitals on a path to a fully paperless, data-driven environment.	8 prescriptive stages (0–7), cumulative requirements. On-site validation for higher stages.
NHS -WGLL [12]	NHS	UK (England)	To provide a common vision for good digital practice and guide NHS trust transformation.	7 “success measures” (capability domains). Used by the regulator (CQC) for assessment.
WHO-PAHO IS4H-Maturity Model [11]	WHO / PAHO	Global / Americas	To strengthen national health systems through improved information and data architecture.	5 maturity levels across domains like Governance, Data Management, and Technology. Self-assessment tool.
Australian Digital Health Capability Framework (ADHCF) [13]	ADHA	Australia	To define and foster the digital health capabilities of the healthcare workforce.	5 domains, 30 capabilities, 3 proficiency levels. Focus on the workforce, not organisations.
JCI Accreditation Standards [3]	JCI	Global	To ensure patient safety and quality of care in accredited healthcare organisations.	Digital health integrated into chapters like ‘Management of Information’. Not a standalone digital model.

### Landscape of digital health maturity models

The review identified a diverse landscape of maturity models, with the most prominent being stage-based, capability-focused frameworks.

#### HIMSS-EMRAM (and related models)

The HIMSS suite of maturity models, particularly the EMRAM, is the most widely cited and adopted framework globally [2]. EMRAM provides a prescriptive 8-stage (0–7) roadmap for hospitals, beginning with the absence of basic systems (Stage 0) and culminating in a fully paperless environment with advanced data analytics and health information exchange capabilities (Stage 7). Each stage has cumulative and specific requirements, covering infrastructure, clinical data repositories, decision support, interoperability, and patient engagement [2]. Its clear, staged approach is a key reason for its widespread international adoption.

#### NHS-WGLL framework

Developed by NHS England, the WGLL framework moves away from a purely prescriptive staged model to a capability-based one. It outlines seven ‘success measures’ for digital transformation: (1) Well-led, (2) Ensure smart foundations, (3) Safe practice, (4) Support people, (5) Empower citizens, (6) Improve care, and (7) Healthy populations. It is used by the Care Quality Commission (CQC), England’s healthcare regulator, to assess how well NHS trusts are embedding digital technology to enhance care [12, 20, 21]. This direct link between a maturity framework and regulatory inspection is a key feature.

#### Other international and LMIC models

Other significant models were identified. The WHO and PAHO have promoted the IS4H maturity model, which focuses on public health systems and data governance [11]. Other frameworks include the ADHCF (workforce focus), the NICE-ESF for digital health technologies, ISO/TS 82,304‑2 quality/label for health apps, and national digital maturity programmes (e.g., Scotland, Germany’s DigitalRadar) [14, 22, 23]. Several reviews highlighted the need for models specifically adapted to LMIC contexts, considering challenges like infrastructure limitations, workforce capacity, and sustainable funding [25, 26, 33, 36, 39–41]. 

### Accreditation-linked digital standards

While maturity models often serve as guidance, accreditation standards make digital proficiency a formal requirement.

#### NABH-DHS (India)

The NABH standards for hospitals and HIS/EMR systems are India’s primary accreditation-linked frameworks [4, 5]. They uniquely combine granular objective elements with a three-tiered maturity assessment (Silver, Gold, Platinum), effectively blending a compliance approach with a developmental roadmap.

#### Other accreditation bodies (JCI, ACHS)

The Joint Commission International (JCI) incorporates information management and technology standards throughout its hospital accreditation program, focusing on continuity of care, data security, and communication [3]. More recently, JCI has launched specific certifications, such as for telehealth, which have dedicated digital standards [42]. ACSQHC embeds digital safety within NSQHS standards; NHS mandates DSP Toolkit compliance against National Data Guardian standards [13, 21]. 

## Crosswalk matrix and gap analysis results

A conceptual crosswalk comparing the NABH chapters to the domains of prominent international models was developed to identify alignment and divergence (Table 3). Alignment strength was determined by the pre-specified anchors described in Methods (Data charting and mapping rules).

**Table 3:** Conceptual crosswalk of international model domains vs. NABH chapters

NABH Digital Health Standard Chapter [4]	HIMSS-EMRAM Alignment [2]	NHS-WGLL Alignment [12]	Alignment with Other Models (WHO, Australia)
Ch. 1: Leadership	Aligns with Stage 7 focus on strategic governance and using data for business/clinical intelligence.	Strong alignment with ‘Well-led’ success measures (vision, strategy, board oversight).	WHO IS4H: Aligns with the ‘Leadership & Governance’ domain. [11]
Ch. 2: Governance	Maps to requirements for policies, procedures, and oversight committees across all stages.	Strong alignment with ‘Well-led’ and aspects of ‘Safe Practice’ (clinical governance).	WHO IS4H: Core alignment with ‘Leadership & Governance’ and ‘Strategy & Investment’ domains.[11]
Ch. 3: Clinical & Patient Safety	Core alignment. Maps to Stage 1 (departmental systems), Stage 4 Computerized Provider Order Entry (CPOE), and Stage 6 (closed-loop medication).	Strong alignment with ‘Safe Practice’ (EPR uptime, clinical safety) and ‘Improve Care’ (decision support).	JCI: Direct alignment with JCI’s focus on patient safety through technology.[3]
Ch. 4: Patient Engagement	Aligns with Stage 7 requirements for a comprehensive patient portal and access to PGHD.	Strong alignment with ‘Empower Citizens’ measure (access to records, shared care plans, self-management tools).	Australian ADHCF: Relates to ‘Information-enabled Care’ capability.[13]
Ch. 5: Information & Data Management	Aligns with Stage 2 (Clinical Data Repository - CDR) and Stage 7 (data warehousing, analytics).	Aligns with ‘Improve Care’ (data for improvement) and ‘Healthy Populations’ (population health data).	WHO IS4H: Aligns with ‘Data Management’ and ‘Analytics’ domains.[11]
Ch. 6: Technology & Infrastructure	Foundational alignment with Stages 0–2 (ancillaries, CDR, basic infrastructure).	Strong alignment with ‘Ensure Smart Foundations’ measure (interoperability, infrastructure, networks).	WHO IS4H: Aligns with ‘Technology’ and ‘Standards & Interoperability’ domains.[11]
Ch. 7: Risk Management	Aligns with security requirements across all stages (e.g., access control, business continuity).	Aligns with ‘Safe Practice’ (cybersecurity). Gap noted: Less explicit on advanced frameworks (e.g., NIST).	JCI: Aligns with standards on data security and privacy in the Management of Information chapter.[3]
Ch. 8: Human Resource Management	Implicitly covered in requirements for training and competency across stages.	Aligns with ‘Support People’ measure (digital literacy, support for workforce).	Australian ADHCF: Strongest alignment. The entire ADHCF is dedicated to this area.[13]

Alignment with Other Models’ aggregates domains from PAHO/WHO IS4H and Australian ADHCF. JCI appears under Clinical & Patient Safety and Risk/Data governance where its ‘Management of Information’ and safety criteria intersect with digital standards; the models are not mutually exclusive, and rows are illustrative of primary alignment

From the mapped evidence, NABH shows strong alignment on governance, clinical documentation/safety, data management, and foundations. The gaps we highlight-AI/algorithm governance, advanced cybersecurity maturity, interoperability maturity metrics, health-equity lens, and PGHD integration - are presented as external guidance areas informed by prominent frameworks and toolkits [16, 17, 19], not as outcomes directly measured within the included studies [14, 16, 18, 27, 28, 34, 37, 38]. 

### Mapping digital health maturity models to position the NABH standards

**Fig. 2:**
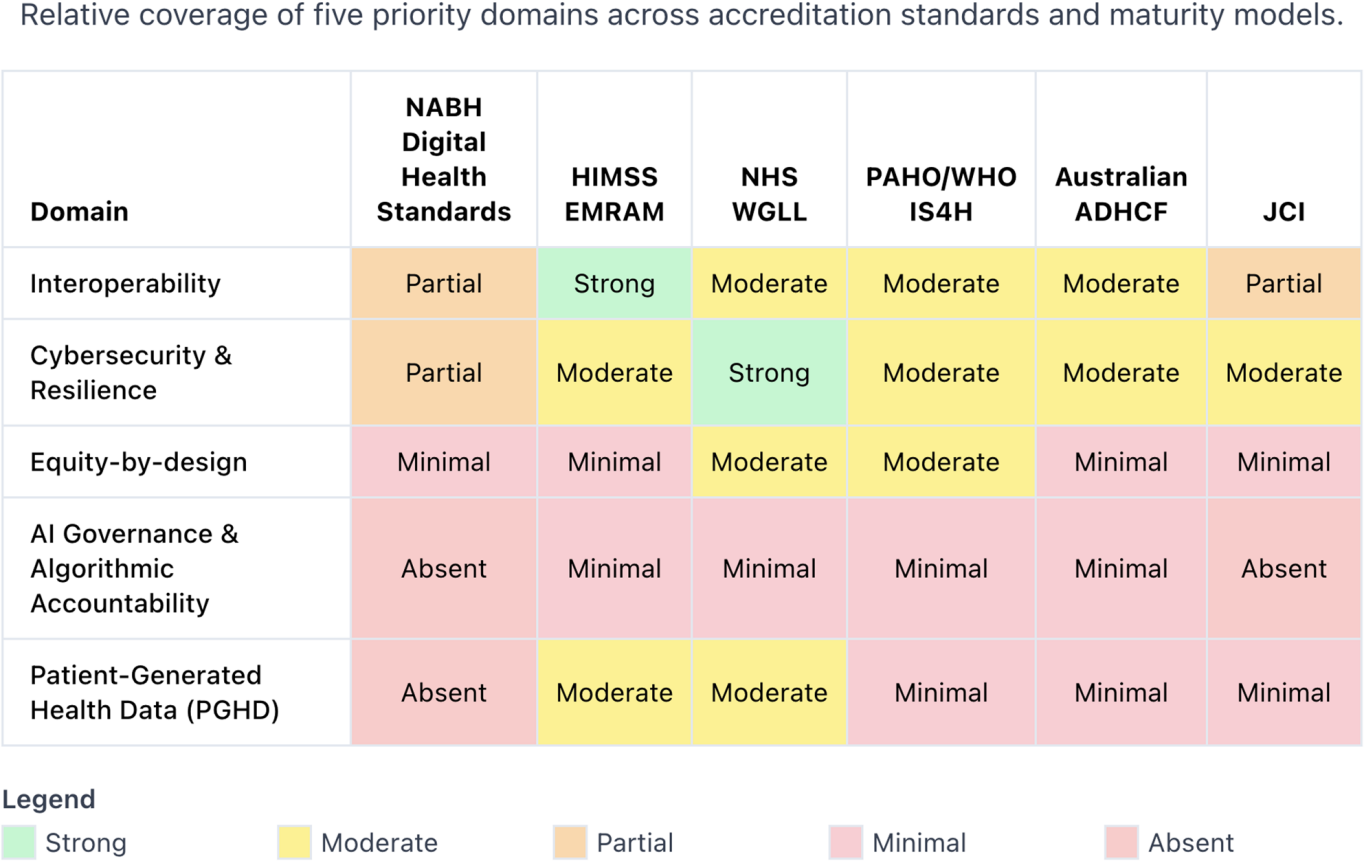
Thematic gap map across NABH, HIMSS EMRAM, NHS WGLL, PAHO/WHO IS4H, Australian ADHCF, and JCI

Global accreditation frameworks have evolved from technology-adoption checklists to holistic socio-technical assurances and, more recently, to special-purpose safeguards. Figure 2 (Thematic Gap Map) summarises how India’s NABH-DHS compare with five influential international models across five priority domains [2, 3, 5, 11, 12, 43]. Cells are colour-coded by relative coverage (green = strong, yellow = moderate, orange = partial, pink = minimal, red = absent), making visible both areas of convergence and domains where NABH criteria could be further strengthened in future iterations.

We treat interoperability and cybersecurity as foundational because they underpin the safe functioning of all other digital capabilities and are already embedded as baseline expectations across several international assurance frameworks. In contrast, equity-by-design, AI governance and systematic PGHD integration are framed as emerging imperatives because they represent newer areas of standard-setting where specific accreditation criteria are still evolving and are less consistently operationalised.

#### Foundational prerequisites

**Interoperability** is now viewed as the bedrock of digital health. Toolkits such as the Health Information Systems Interoperability Maturity Toolkit help organisations move from basic data exchange to strategic, system‑wide interoperability [18]. 

**Cybersecurity** is no longer optional. The NIST CSF 2.0 sets a six‑function lifecycle (Govern, Identify, Protect, Detect, Respond, Recover) that many countries adopt wholesale [16]. 

#### Emerging imperatives

##### Health equity

Digital tools can widen or narrow disparities. Equity‑by‑design checks‑lists based on the socio‑ecological model are only beginning to appear, and most standards-NABH included-lack measurable criteria.

##### AI governance

With AI moving from pilots to routine care, the new ISO/IEC 42,001 offers a certifiable management system covering risk, ethics and lifecycle oversight; Utilization Review Accreditation Commission (URAC) and others are launching AI‑specific accreditations [17, 35, 44–48]. 

##### Patient‑generated health data (PGHD)

Wearables and remote monitoring demand guidance on validation, clinical integration and liability-areas most legacy standards overlook.

#### Gap analysis for NABH

A concise summary of prioritized gaps and illustrative external references is provided in Table 4.

**Table 4:** Gap analysis for NABH

Priority gap [4]	Why it matters	Illustrative global reference
AI governance	Ensures safe, bias-free clinical AI	ISO/IEC 42,001; URAC AI programme [17]
Advanced cybersecurity	Moves from passive controls to proactive defence	NIST CSF 2.0; incident-response simulations [16]
Interoperability maturity metrics	Tracks progress beyond “use standards” tick-boxes	Interoperability Maturity Toolkit [18]
Health-equity lens	Prevents digital divide in access and outcomes	WHO/ITU equity guidance [24]
PGHD integration	Unlocks remote care and precision public health	FDA/IMDRF real-world-data guides [28]

#### Evolutionary trajectory

1. Evolution of digital assurance (indicative milestones).
2. Adoption era (2005–2015): staged EHR maturity (HIMSS EMRAM) catalysed core digitization [2].
3. System-thinking era (2016–2021): capability-led maturity embedded into regulation (e.g., NHS WGLL used by CQC). (NHS England, 2021/2023) [12].
4. Assurance baseline (2022–2024): cybersecurity and interoperability established as universal prerequisites (NIST CSF 2.0; Interoperability Maturity Toolkit) [16, 18].
5. Societal safeguards (2023–present): AI governance and equity-by-design enter standards discourse (ISO/IEC 42001) [17].

#### Implications for India

NABH already anchors risk management and basic interoperability but must embed granular requirements in the five gap domains above to remain future‑proof. Adopting a ‘maturity ladder’ for each theme-mirroring the international trajectory-will let Indian hospitals benchmark progress, align with emerging global certifications and, importantly, safeguard patient trust as digital health scales.

### Implementation guidance and challenges

Recurring challenges in LMIC settings include sustained financing, infrastructure deficits, workforce capacity, and weak data‑use culture. Success correlates with strong mandates, public–private partnerships, and incremental, context‑aware improvement. The World Bank’s ‘Digital‑in‑Health’ proposes a Prioritize–Connect–Scale approach, complementing WHO’s Global Digital Health Strategy [24, 30, 39]. 

## Discussion

### Overview and interpretation

This scoping review maps where the NABH-DHS converge with widely used digital assurance and capability frameworks and where important opportunities remain. Strengths of this scoping review include a comprehensive multi‑source search and adherence to PRISMA‑ScR. The results established strong alignment on governance, clinical documentation and safety, information and data management, and foundational technology. Building on those findings, this discussion interprets what those alignments mean for implementation in Indian hospitals and how policy makers can use the mapped gaps-particularly cybersecurity maturity, measurable interoperability, AI governance, equity, and PGHD-to guide the next iteration of NABH-DHS without overstating the underlying evidence.

### What the alignments enable now

#### Assurance of core digital capability

Convergence between NABH-DHS and maturity/assurance frameworks indicates that hospitals can rely on the current DHS to drive core EMR/HIS functionality, data quality controls, clinical documentation safety, and baseline infrastructure. From a purchaser and regulator perspective, this reduces variability in minimum digital standards and provides a credible platform for incremental progression.

#### A pragmatic hybrid for diverse settings

The DHS blends compliance‑style requirements (surveyable, binary) with an implicit maturity path (tiered progression). This hybrid is particularly suitable for India’s mixed public–private system and variable digital baselines. It allows small and mid‑size hospitals to achieve compliance while signalling a trajectory toward more advanced capabilities as capacity and resources grow.

#### Regulatory coherence

Areas of strong alignment (e.g., leadership and governance, information management, safety processes) provide an immediate opportunity for coherence across accreditation, state health insurance empanelment, and programme audits. Using DHS as the common denominator can reduce duplicate checks and lower administrative burden for providers.

### Priority opportunities: how to translate ‘gaps’ into implementable guidance [16, 18, 22, 27, 28, 44, 45]

The mapped ‘gaps’ are best treated as external guidance to harden future editions of DHS while keeping the current compliance pathway intact. Below we articulate actionable options and success indicators that policy makers and hospitals can adopt without requiring a wholesale redesign of the DHS.

#### Cybersecurity maturity

Add a short set of tiered, outcome‑oriented attestations that reference an established cybersecurity framework (e.g., governance function, risk assessment cadence, incident response testing). *Policy signal*: require annual self‑attestation plus evidence of one completed tabletop exercise. *Provider indicator*: documented risk register with treatment actions and post‑incident review within 30 days of any material event.

#### Interoperability-measure what matters

Move from capability statements to maturity metrics that capture use and outcomes (e.g., percentage of discharge summaries exchanged using nationally adopted standards; time to successful external record retrieval). *Policy signal*: tie incremental incentives or fast‑track empanelment to improvement in these metrics. *Provider indicator*: year‑on‑year rise in standards‑based exchanges and reduced repeat tests across care transitions.

#### AI lifecycle governance

Rather than creating a new chapter, add a concise governance control set covering: use‑case registration, data provenance/quality checks, validation prior to deployment, human‑in‑the‑loop safeguards, monitoring for drift, and decommissioning. *Policy signal*: hospitals maintain an AI use‑case registry approved by a multidisciplinary committee. *Provider indicator*: model cards or equivalent artefacts on file; documented performance monitoring with thresholds for suspension.

#### Equity and inclusion checks

Embed light‑weight, auditable prompts where digital processes materially affect access (e.g., patient portals, telehealth). *Policy signal*: require an annual accessibility review and patient advisory input. *Provider indicator*: availability of alternative access pathways; tracked uptake by priority populations; remediation log for identified barriers.

#### Patient‑generated health data (PGHD)

Provide guidance for validating, attributing, and acting on PGHD within clinical workflows. *Policy signal*: clarify when PGHD may be used for decision support and what validation is needed. *Provider indicator*: documented protocols for device/app verification and reconciliation into the record with clinician acknowledgment.

### Implications for NABH: low‑friction ways to incorporate guidance [16, 18, 22, 27, 28, 34, 44, 45]

#### Footnote‑level additions over chapter rewrites

Introduce a handful of auditable statements and example indicators in annexes/FAQs rather than altering chapter counts or sequence. This preserves surveyor training investments and provider preparedness while signalling forward compatibility.

#### Phased progression via tiers

Keep the Silver/Gold/Platinum trajectory but associate optional ‘digital plus’ badges with achievement on the five guidance areas. This maintains inclusivity for smaller hospitals while rewarding leaders.

#### Reference, not replicate

Where external frameworks are mature (e.g., cybersecurity, interoperability toolkits), reference them explicitly and require evidence of use, avoiding parallel, bespoke checklists that fragment conformance.

### Implications for hospitals and vendors [2, 12, 21, 27]

Hospitals can use the crosswalk matrix as a roadmap: first close partial areas that are already surveyable (risk registers, workforce training, patient engagement artefacts), then pilot indicators for interoperability and cybersecurity that are feasible with existing systems. A simple internal dashboard covering the five guidance domains will make accreditation visits smoother and support board oversight.

Vendors should align release notes and implementation guides to DHS language (e.g., mapping features to DHS objectives). Where AI features are offered, provide model documentation and monitoring hooks that hospitals can insert into their AI registry and governance process.

### Policy considerations for scale and equity

National or state programmes can leverage the DHS to drive **consistency with flexibility**: set minimums for all providers, then allow states or payer schemes to add one or two context‑specific indicators (e.g., regional referral exchange or telehealth accessibility) without altering the national core. Importantly, interoperability metrics and equity checks should be collected in a way that does **not** penalize facilities serving higher‑risk or under‑resourced populations-reporting and improvement support should precede sanctions. For capacity‑constrained settings, a ‘shared services’ model (regional incident‑response exercises; shared interoperability testing sandboxes) can reduce the burden on small hospitals while raising the floor system‑wide.

### Relationship to existing literature and frameworks

Our interpretation is consistent with how capability frameworks have evolved globally: staged EMR adoption established the digital foundation; whole‑organization capability models clarified leadership, workforce, and citizen‑empowerment requirements; more recently, cybersecurity, interoperability outcomes, AI governance, and equity have emerged as necessary safeguards. We intentionally treat these latter themes as **guidance** rather than as findings from the included studies to preserve methodological integrity.

## Limitations of the review

As a scoping review, this work does not estimate effects; alignment ratings are interpretive despite dual review and consensus procedures. English‑language restriction may under‑represent local innovations. We also did not complete stakeholder consultations; hence, the policy options we outline should be road‑tested with implementers and surveyors before widespread adoption. We partially mitigated mapping subjectivity by using a piloted charting template, dual independent mapping with consensus and additional-reviewer adjudication, but some interpretive judgement inevitably remains. Future research priorities include empirical evaluation of NABH standards’ impact, economic analyses of maturity levels, and India‑specific methods for AI governance and digital health equity.

## Conclusion

The NABH Digital Health Standards align with major international domains, providing a robust foundation for India’s digital transformation. Policymakers should leverage the current DHS to lock in core digital assurance while simultaneously signalling a pragmatic next step: requiring light-weight artefacts and indicators for cybersecurity maturity, measurable interoperability, AI lifecycle governance, equity, and PGHD. Implementing these through annexes, registries, and badges - rather than immediate chapter rewrites - offers a scalable ‘Digital Plus’ model for other emerging economies. This approach secures patient safety and data trust today, while building a pathway for hospitals to progress toward a learning, equitable, and resilient digital health system.

## Supplementary Information

Below is the link to the electronic supplementary material.

Supplementary Material 1

## Data Availability

The datasets (charting forms and evidence tables) generated and analysed during the current study, together with the full search strategies and other supplementary materials, are available from the corresponding author on reasonable request.

## Abbreviations

ACHS: Australian Council on Healthcare Standards
ACSQHC: Australian Commission on Safety and Quality in Health Care
ADHCF: Australian Digital Health Capability Framework
AI: Artificial Intelligence
CQC: Care Quality Commission
CSF: Cybersecurity Framework
DSP: Data Security and Protection
EMRAM: Electronic Medical Record Adoption Model
HIS: Hospital Information System
IMDRF: International Medical Device Regulators Forum
IS4H: Information Systems for Health
JCI: Joint Commission International
LMIC: Low- and Middle-Income Countries
NABH: National Accreditation Board for Hospitals & Healthcare Providers
NIST: National Institute of Standards and Technology
ONC: Office of the National Coordinator for Health IT
PGHD: Patient-Generated Health Data
RWD/RWE: Real-World Data/Evidence
WGLL: What Good Looks Like
