# Supplementary material for: Mapping digital health maturity models and accreditation-linked standards: a scoping review to position the National Accreditation Board for Hospitals & Healthcare Providers (NABH) digital health standards of India
Source: BMC Health Serv Res. 2026 Mar 11;26:426. doi: 10.1186/s12913-026-14301-y (PMC13032403; doi:10.1186/s12913-026-14301-y)
Supplement: Supplementary file 1 — Supplementary Material 1 [file 12913_2026_14301_MOESM1_ESM.docx]

# Supplementary Materials / Appendices

## S1. Full database search strategies (last accessed on 26th July, 2025)

### PubMed Search Strategy

(("digital health"[Title/Abstract] OR "digital maturity"[Title/Abstract] OR "digital health maturity"[Title/Abstract] OR "maturity model"[Title/Abstract] OR "maturity assessment"[Title/Abstract]) AND ("health"[Mesh] OR "health information systems"[Mesh] OR "medical informatics"[Mesh]))

("digital health"[Title/Abstract] OR "digital maturity"[Title/Abstract] OR "digital health maturity"[Title/Abstract] OR "maturity model"[Title/Abstract] OR "maturity assessment"[Title/Abstract]) AND ("health"[MeSH Terms] OR "health information systems"[MeSH Terms] OR "medical informatics"[MeSH Terms])

### Scopus Search Strategy

(TITLE-ABS-KEY("digital health" OR "health information system*" OR "medical informatics" OR "eHealth" OR "mHealth") AND TITLE-ABS-KEY("maturity model*" OR "accreditation standard*" OR "capability framework" OR "assessment framework" OR "quality assurance"))

### Embase Search Strategy

(('digital health':ti,ab OR 'digital maturity':ti,ab OR 'digital health maturity':ti,ab OR 'maturity model':ti,ab OR 'maturity assessment':ti,ab OR 'maturity framework':ti,ab OR 'digital transformation':ti,ab OR 'digital readiness':ti,ab OR 'e-health maturity':ti,ab OR 'ehealth maturity':ti,ab) AND ('health information system'/exp OR 'medical informatics'/exp OR 'health care'/exp OR 'hospital'/exp OR 'health care quality'/exp OR 'health care organization'/exp OR 'electronic health record'/exp OR 'telemedicine'/exp OR 'health technology'/exp)) OR (('maturity model':ti,ab OR 'maturity assessment':ti,ab OR 'assessment framework':ti,ab OR 'evaluation framework':ti,ab) AND ('accreditation':ti,ab OR 'quality assurance':ti,ab OR 'standard'/exp OR 'benchmarking':ti,ab OR 'quality indicator'/exp OR 'performance measurement'/exp) AND ('health care'/exp OR 'hospital'/exp OR 'health information system'/exp)) AND [english]/lim AND ('article'/it OR 'review'/it)

### Grey Literature Search Strategy (Google/Google Scholar)

"Digital health maturity" "maturity model" site:.gov OR site:.org OR site:.int

"Digital health maturity assessment" filetype:pdf

# S2. SWiM (Synthesis Without Meta‑analysis) Reporting Checklist

| **Section** | **#** | **SWiM Item** | **Where Reported in Manuscript** |
| --- | --- | --- | --- |
| METHODS | 1 | Grouping of studies: Explain how studies were sorted. | Methods → Synthesis plan (narrative synthesis, crosswalk, gap map) |
|  | 2 | Standardised metric: Describe common measures used. | Methods → Synthesis plan; Results → Characteristics of included sources (descriptive numerical summary; counts of source types/origins) |
|  | 3 | Synthesis method: Detail how results were combined. | Methods → Synthesis plan (narrative synthesis, conceptual crosswalk matrix, thematic gap map) |
|  | 4 | Criteria to prioritise results: State rules for highlighting. | Methods → Synthesis plan (priority gap domains identified by triangulation) |
|  | 5 | Exploration of heterogeneity: Methods to explore differences. | Not applicable (descriptive scoping review) |
|  | 6 | Certainty in evidence: Approach to assess confidence. | Methods → Critical appraisal (formal appraisal not undertaken) |
| RESULTS | 7 | Summary of synthesis findings: Present main combined results. | Results → Crosswalk Matrix and Gap Analysis Results; Results → Mapping Digital Health Maturity Models...; Table 3; Figure 2; Table 4 |
|  | 8 | Limitations of the synthesis: Discuss synthesis-level limitations. | Discussion → Limitations of the synthesis |
|  | 9 | Implications: For practice, policy, or research. | Discussion → Implications for NABH; Discussion → Implications for hospitals and vendors; Discussion → Policy considerations for scale and equity; Discussion → Future research priorities |
